# Therapies for diabetic gastroparesis: A protocol for a systematic review and network meta-analysis

**DOI:** 10.1097/MD.0000000000020461

**Published:** 2020-05-22

**Authors:** Shengju Wang, Ruili Wang, Yanli Zhang, Xu Zhang, Baochao Cai, Yan Lu, Yuguo Xia, Qiu Chen

**Affiliations:** a Department of Endocrinology, Hospital of Chengdu University of Traditional Chinese Medicine; b Diabetes Department, Jintang County Traditional Chinese Medicine Hospital, Chengdu; c Endocrinology Department, Jiaxing Hospital of Traditional Chinese Medicine, Jiaxing; d Department of Endocrinology, Third Affiliated Hospital of Chengdu University of TCM, Chengdu, China.

**Keywords:** diabetic gastroparesis, network meta-analysis, protocol, systematic review

## Abstract

**Background:**

Diabetic gastroparesis (DG) is a common autonomic neuropathy which impacts on nutritional state and quality of life in diabetic patients, and it also adversely affects glycemic control in diabetes. The prevalence of DG is growing with the number of patients with diabetes continues to increase. However, there is no definitive answer as to which of the current therapies is the best for the clinical treatment of the different manifestations of DG. The subject of this study is to answer the following question: what is the best intervention for diabetic patients with gastroparesis?

**Methods:**

Comprehensive searches of the Cochrane Library, PubMed, Embase, Medline, Central and Web of Science, and 4 Chinese databases, including China National Knowledge Infrastructure, VIP Database for Chinese Technical Periodicals, Chinese Biomedical Literature Database, and WanFang will be completed using the following keywords DG and therapies and related entry terms. Studies will be included, according to the eligibility criteria (randomized controlled trials and controlled clinical trials, considering specific outcome measures for DG). The reference lists of included studies will be manual searched. Relevant data will be extracted from included studies using a specially designed data extraction sheet. Risk of bias of the included studies will be assessed, and the overall strength of the evidence will be summarized through GRADE. A random effects model will be used for all pairwise meta-analyses (with a 95% confidence interval). A Bayesian network meta-analysis will explore the relative benefits between the various therapies. The review will be reported using the Preferred Reporting Items for Systematic Reviews incorporating Network Meta-Analyses statement. Network meta-analysis will be performed using a Bayesian framework through the Winbugs software.

**Results:**

This network meta-analysis will identify the best effective therapy for DG.

**Conclusion:**

This study will compare and evaluate current therapies for DG, and find the best treatment of DG.

## 1 Introduction

Gastroparesis is defined as a delay in the emptying of ingested food in the absence of obvious structural abnormalities of the stomach or duodenum.^[1]^ The most common cause of gastroparesis is diabetes mellitus.^[2]^ The prevalence of diabetic gastroparesis (DG) is growing with the number of diabetic patients continues to increase. Diabetic patients with gastroparesis often have many of the chronic complications of diabetes and increased hospitalization. In some patients, gastroparesis is the chief diabetic, neuropathic complication. Mortality is increased in diabetic patients when they develop gastroparesis and is usually related to cardiovascular events when compared with diabetic patients without gastroparesis.^[3]^

The DG can manifest in a variety of symptoms, including early satiety, chronic nausea and vomiting, anorexia, abdominal distention and pain, postprandial fullness.^[4]^ DG can be associated with anxiety and depression, can impair quality of life, and can impact on self-management of diabetes, especially patients with fluctuation of blood glucose.^[5,6]^ In diabetic patients with gastroparesis, ingested food is not emptied in an appropriate term of time; thus, the nutrient absorption is abnormal. Consequently, the selected dose and timing of insulin treatment to control postprandial glucose may be inappropriate.

In many patients with gastroparesis, unstable postprandial glucose concentrations result in amplitudes from hypoglycemia to severe hyperglycemia and even ketoacidosis.^[7]^ Hyperglycemia itself induces gastric dysrhythmias and slows gastric emptying.^[8]^

The DG is primarily a disease of gastric enteric neurons and interstitial cells of Cajal.^[9,10]^ Interstitial cells of Cajal is depleted in the DG stomach, which contributes to gastric emptying aberrations.^[9,11,12]^ Gastric enteric neurons are decreased in numbers of cell bodies and processes are truncated. The immune system and carbon monoxide are also played a role in the pathogenesis of DG.^[13]^ The circular and longitudinal smooth muscle layers are normal or have very mild fibrosis.

Stabilize diabetes control are the basal managements for DG. Meanwhile, there are several therapies have been used to improve the rate of gastric emptying for treatment of DG, they are including medicine therapies, electric therapies, endoscopic therapies, and diet therapies. Medicine therapies encompass prokinetic therapy (Macrolides, substituted Benzamides) and antinauseant therapy (Serotonin Antagonists, phenothiazines, Butyrophenones, Benzodiazepines). Prokinetic agents are applied to ameliorate the rate of gastric emptying have not improved the symptoms related to DG. Antiemetics are used as first-line therapy. Although these agents decrease symptoms and improve nutritional intake, their use is associated with adverse effects. Dietary modification is beneficial but infrequently followed.^[14,15]^ Gastric electrical stimulation can improve symptoms and reduce morbidity for a long-term follow-up, but there is weight gain in some patients.^[16,17]^

Accumulating evidence shown that traditional Chinese medicine and acupuncture may be beneficial for treating DG, once the limited efficacy of the medicine therapies and when patients cannot tolerate their side effects. It has been demonstrated that traditional Chinese medicine and acupuncture can improve gastric motility and facilitate gastric emptying in human.^[18–20]^ Other therapies are also used to treat DG, including botulinum toxin, transpyloric stenting, and gastric per-oral endoscopic myotomy. Surgical treatments (laparoscopic pyloroplasty, gastrectomy, antrectomy, and pancreatic transplantation) are used in severe refractory DG. They are the last options for treating DG.

Treatments in patients diagnosed as DG needed to decrease symptoms, improve quality of life, ensure enough nutritional intake, and control progression of DG. These signs and symptoms can be caused or even deteriorate by DG, but the current support therapies for DG are mainly controlling the consequences rather than tackling the causes of these problems. The multifactorial etiology of DG makes these problems are intractable by the clinicians.

## 2 Review aims

We plan to perform a systematic review and network meta-analysis to attempt to answer the following question: which is the best treatment for diabetic patients with gastroparesis, considering the improvement of symptoms, quality of life, nutritional intake, complications, and costs of the different therapies found. We will explore in this review as primary outcome parameter is symptom scoring and grading of severity by Patient Assessment of Upper Gastrointestinal Disorders Symptom Severity Index (PAGI-SYM) questionnaire^[21]^ or Gastroparesis Cardinal Symptom Index (GCSI),^[22]^ gastric emptying by scintigraphy or radio-opaque markers,^[23,24]^ and other testing tools for gastroparesis and gastric dysrhythmias (wireless capsule motility test, electrogastrography). The secondary outcome measurement was the quality of life by Patient Assessment of Upper Gastrointestinal Disorders-Quality of Life (PAGI-QOL).^[25]^ The psychological measurements by Beck Depression Inventory (BDI)^[26]^ and State-Trait Anxiety Inventory (STAI)^[27]^ are also implore in this review.

## 3 Why it is important to do this review

The DG is a common autonomic neuropathy which impacts on nutritional state and quality of life in diabetic patients, it also adversely affects glycemic control in diabetes. There still lacks an effective long-term therapy to treat DG, and a few remaining questions still need to be tackled, such as “what should be done when the side effects of current therapies are overwhelming to patients” or “what should be done when the symptoms are still unimproved after treating with current therapies” or “is there an alternative potential effective therapy to treat the causes of DG.” There still lack of evidences to answer these problems definitively. Several reviews, systematic reviews and meta-analysis are only compared 2 treatments at a time, not summarized a more comprehensive set of comparisons tackling the multiple treatments available. However, some studies described different current available interventions.^[4,28,29]^ It would be helpful for the clinician and patients to know their advantages and disadvantages to better choose the current available therapies for the different manifestations of DG.

## 4 Methods

The protocol of this systematic review and network meta-analysis will be written in accordance with the Preferred Reporting Items for Systematic Review and Meta-Analysis Protocols (PRISMA-P)^[30]^ guidance. The completed systematic review will be written using the Preferred Reporting Items for Systematic Reviews Incorporating Network Meta-Analyses^[31]^ extension statement to structure the contents of the final report. This protocol is registered in the INPLASY database (https://inplasy.com/inplasy-2020-4-0176/) and INPLASY registration number is INPLASY202040176. The literature search was established to answer the research question phrased as follows in the PICO framework: population: adult diabetic patients diagnosed with DG; interventions and comparisons: medication (Macrolides, Substituted Benzamides, Serotonin Antagonists, phenothiazines, Antihistamines, Butyrophenones, Antidepressants, Benzodiazepines), venting gastrostomy, jejunostomy, transpyloric stenting, gastric per-oral endoscopic myotomy, compared among them or to placebo; botulinum toxin intrapyloric injections compared to placebo or other therapies, surgical treatments (laparoscopic pyloroplasty, gastrectomy, antrectomy, pancreatic transplantation), gastric electrical stimulation, traditional Chinese medicine, acupuncture (electroacupuncture, scalp acupuncture, eye acupuncture, abdomen acupuncture, ear acupuncture). It is possible in some articles that the therapies’ results might be compared among them, compared to placebo groups or to controls; outcomes: reduction of symptoms (e.g., early satiety, fullness, nausea, vomiting, anorexia, bloating, abdominal pain, either self-reported or through PAGI-SYM and GCSI), improvement of gastric emptying (through scintigraphy or radio-opaque markers or wireless capsule motility test or electrogastrography), and the quality of life (through PAGI-QOL), the psychological measurements (depression, anxiety, through BDI and STAI); study design: randomized controlled trials (RCTs) and controlled clinical trials (CCTs). Published trial will be from inception to May 2021 in the English and Chinese language.

## 5 Criteria for selecting studies for this review

### 5.1 Types of participants

Studies that enrolled patients (from 18 years of age, regardless of gender and ethnicity) were diagnosed as diabetes with dyspeptic symptoms excluding gastric outlet obstruction or ulceration.

### 5.2 Interventions

According to the literature, treatments for DG are of wide variation and can be split into the following groups: medicine therapies: Macrolides, Substituted Benzamides, Serotonin Antagonists, phenothiazines, Antihistamines, Butyrophenones, Antidepressants, Benzodiazepines; endoscopic therapies: botulinum toxin A intrapyloric injections, balloon dilation of pylorus, radiofrequency ablation at lower esophageal sphincter; electric therapies: gastric electric stimulation, gastric pacing; diet therapies: gastroparesis diet, high-protein drinks, total parenteral nutrition; traditional Chinese medicine: Xiangshaliujunzi Decoction; acupuncture: electroacupuncture, scalp acupuncture, eye acupuncture, abdomen acupuncture, ear acupuncture; surgical therapies (laparoscopic pyloroplasty, gastrectomy, antrectomy, transpyloric stenting, gastric per-oral endoscopic myotomy, pancreatic transplantation); and others: alternative or support therapies. Any study evaluating any of the treatments listed above will be kept for inclusion in this review. Analysis will be performed at therapy/group level.

### 5.3 Types of studies

We will include RCTs and CCTs. Review papers, expert opinions, case reports, and series of case reports will be excluded. The literatures of relevant systematic reviews will be viewed to identify any studies missed by our literature search.

## 6 Information sources and literature search

### 6.1 Electronic searches

Comprehensive searches of the Cochrane Library, PubMed, Embase, Medline, Central and Web of Science, and 4 Chinese databases, including China National Knowledge Infrastructure, VIP Database for Chinese Technical Periodicals, Chinese Biomedical Literature Database, and WanFang will be completed using the strategy described in Table 1, adjusting different search methods according to different Chinese and English databases. Two independent authors will select the literatures. The search will include all the indexed papers, computerized literature databases supplemented by hand searching of reference lists from each relevant article identified.

**Table 1:**
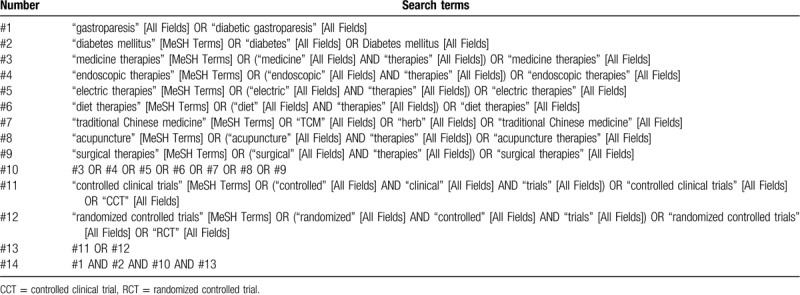
Example of PubMed search strategy.

### 6.2 Searching other resources

At the same time, we will retrieve other resources to complete the deficiencies of the electronic databases, mainly searching for the clinical trial registries and grey literature about treatments for DG on the corresponding website.

### 6.3 Study selection procedure

**Figure 1:**
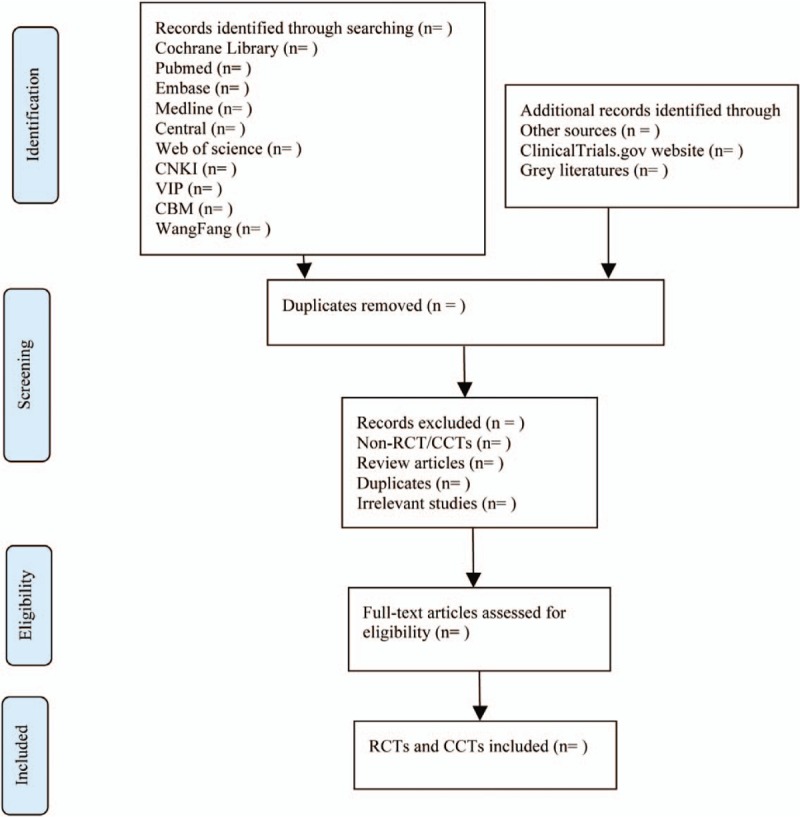
Flow chart of the study selection. RCT = randomized controlled trial.

All titles and abstracts found will be independently read. After the searches, when found, the duplicates will be removed and the articles evaluated. The abstracts found in multiple searches to identify potentially eligible papers for containing will be also studied. Inconsistencies will be addressed by discussion among independent researchers. In case of missing data or information, authors will be contacted. The reviewers that will be enrolled in the searches are experienced in diabetes management, specialist clinicians, or methodologists in evidence-based medicine. PRISMA flow diagram (Fig. 1) will be used to show the screening process of the study.

### 6.4 Data collection process

A standardized, electronic data collection form implemented in Microsoft Excel will be applied to extract the following data: study design, diagnosis, number of participants, types of therapies compared, patient demographics, outcome measures, results, risk of bias assessment, and study authors’ main conclusions. Two investigators will conduct data extraction independently.

### 6.5 Outcomes

Different outcomes will be considered in this review whenever available:

- Primary effects: reduction of symptoms (through self-reported or detected by PAGI-SYM or GCSI), improvement of gastric emptying (through scintigraphy or radio-opaque markers or wireless capsule motility test or electrogastrography)
- Secondary effects: the quality of life (through PAGI-QOL), the psychological measurements (through BDI and STAI)
- Comorbidity (side effects or adverse events), costs, time span of the intervention (short-span and long-span outcomes can be evaluated separately)
- Compliance (adherence to the intervention) with the different therapies

### 6.6 Heterogeneity assessment

The different manifestations of DG may be treated separately in the outcome analysis, considering the subgroup analyses and/or meta-regression.

### 6.7 Assessment of effectiveness

The tools used to verify the effectiveness of DG therapy are mostly PAGI-SYM or GCSI, scintigraphy or radio-opaque markers or wireless capsule motility test or electrogastrography, PAGI-QOL. Different evaluation methods are selected according to the different efficacy indicators.

### 6.8 GRADE assessment

The evidence will be interpreted according to the GRADE Working Group method for rating the quality of intervention effect evaluates from network meta-analysis. This approach is based on 4 steps considering direct and indirect intervention evaluations for each comparison of the evidence network, rating the quality of each direct and indirect effect assessment, rating the network meta-analysis evaluate for each comparison of the evidence network and quality of each network meta-analysis effect assessment.^[32]^

### 6.9 Risk of bias assessment

Studies will be evaluated for bias using the Cochrane risk of bias tool considering the judgment of the random sequence generation, allocation concealment, blinding of participants and personnel, blinding of outcome estimation, incomplete outcome data, selective reporting, and other sources of bias as “Low risk” of bias, “High risk” of bias, or “Unclear risk” of bias.

### 6.10 Data synthesis

An overview of all selected researches will be narratively exhibited. Once data are obtained, a sheet will be made to tabulate data for the different outcomes. Classification according to the population and study characteristics and nature of the therapy will be made. Both traditional pairwise meta-analyses and network meta-analyses will be performed.

### 6.11 Standard pairwise meta-analysis

A random effects model will be used for all pairwise analyses when data are available. The heterogeneity will be assessed through the evaluation of the variance between researches (Chi-squared test and *I*^2^ statistic). We will divide the treatment efficacy into significantly effective, effective, and ineffective, and the number of people who were in ineffective group will be used to calculate the odds ratios and corresponding 95% confidence intervals. Mean differences or standard mean difference between treatments may also be considered.

### 6.12 Network geometry

The network of therapies will be judged based on the available study data presented and assessed graphically. We will estimate if there is a sufficient number of comparisons in network with no available data, if there is a high number of comparisons based on single studies, if there are any “closed loops” which allow testing agreement between direct and indirect assessments for comparison on network, if any key interventions are missing, and if the possible lumping of therapies is minimizing the clinical relevance of the review. Next, the feasibility of a network meta-analysis will be evaluated. Interventions will possibly be lumped into eight groups a priori considering the type of therapy: medicine therapies, endoscopic therapies, electric therapies, diet therapies, traditional Chinese medicine, acupuncture, surgical treatments, and other alternative or support therapies.

### 6.13 Network meta-analysis

Network meta-analysis will be conducted using a Bayesian framework through the Winbugs software considering the random effects models, which use vague prior distributions for all therapy effects as well as the between-study variance parameter. The results of all pairwise comparisons will be reported as odds ratio and corresponding 95% credibility intervals. The median interventions rankings and the surface under the cumulative ranking curve will be displayed as well. Analyses will be conducted using Markov chain Monte Carlo approaches.

We will evaluate the convergence based on the Gelman Rubin diagnostics and inspection of Monte Carlo errors.^[33]^ The consistency of results will be estimated, monitoring through the comparison of results of pairwise and network meta-analyses. Also, we will assess the consistency by fitting the consistency and inconsistency models for network meta-analyses and through the comparison of deviance information criterion between both models with smaller values indicative of a better fit and considering a difference of 5 or more as important.^[34]^ Possible violation of transitivity could be related to containing of subjects with different states of health and different habits. We will investigate this through a subgroup and meta-regression analyses considering the following factors: inclusion of patients with overweight, poor compliance, alcohol drinkers or a history of drinking, caffeine drinkers, smokers or a history of smoking, adults with a highly stressful life.

### 6.14 Sensitivity analysis

Sensitivity analysis is mainly used to assess the robustness of the primary outcome measures. The approach is that removing the low-level quality study one by one and then merging the data to evaluate the impact of sample size, study quality, statistical method, and missing data on results of network meta-analysis and traditional meta-analysis.

## 7 Discussion

The DG is an intractable complication of diabetes and increasingly being recognized as a significant health problem. The treatment of DG is difficult because of its multifactorial etiology and fluctuating symptoms. The efficacy of current treatments is limited. This network meta-analysis is performed to identify and evaluate therapies to treat DG. Although the comparison among therapies in a network meta-analysis is conducted based on direct comparisons of treatments and indirect comparisons based on a common measurement, some factors may affect the outcomes obtained from the analysis as number of articles included in the network; heterogeneity and inconsistency and several approaches have been applied to tackle those factors.^[35,36]^

In conclusion, this systematic review will compare current therapies for DG, and find the best treatment of DG. We hope this study may lead to some recommendations, for both patients and investigators, as which is the best treatment for a specific patient case and how future researches need to be designed, considering what is available now and what is the reality of the patient.

## Author contributions

**Conceptualization:** Shengju Wang, Ruili Wang.

**Data curation:** Shengju Wang, Ruili Wang, Xu Zhang.

**Formal analysis:** Yanli Zhang, Baochao Cai, Yan Lu.

**Funding acquisition:** Yuguo Xia.

**Investigation:** Shengju Wang, Ruili Wang, Yuguo Xia, Qiu Chen.

**Methodology:** Shengju Wang, Ruili Wang, Xu Zhang.

**Project administration:** Yan Lu.

**Resources:** Shengju Wang, Ruili Wang, Yanli Zhang.

**Software:** Shengju Wang, Xu Zhang, Baochao Cai.

**Supervision:** Yuguo Xia, Qiu Chen.

**Validation:** Qiu Chen.

**Writing – original draft:** Shengju Wang, Ruili Wang.

**Writing – review & editing:** Yuguo Xia, Qiu Chen.
